# Editorial: Multiscale Modeling From Macromolecules to Cell: Opportunities and Challenges of Biomolecular Simulations

**DOI:** 10.3389/fmolb.2020.00194

**Published:** 2020-08-28

**Authors:** Giulia Palermo, Alexandre M. J. J. Bonvin, Matteo Dal Peraro, Rommie E. Amaro, Valentina Tozzini

**Affiliations:** ^1^Departments of Bioengineering and Chemistry, University of California, Riverside, Riverside, CA, United States; ^2^Faculty of Science - Chemistry, Bijvoet Centre for Biomolecular Research, Utrecht University, Utrecht, Netherlands; ^3^Institute of Bioengineering, School of Life Sciences, École Polytechnique Fdédérale de Lausanne, Lausanne, Switzerland; ^4^Department of Chemistry and Biochemistry, University of California, San Diego, La Jolla, CA, United States; ^5^Istituto Nanoscienze, CNR, Pisa, Italy; ^6^Lab NEST, Scuola Normale Superiore, Pisa, Italy

**Keywords:** multiscale modeling, molecular dynamics simulations, advanced sampling methods, coarse grained models, macro-biomolecules, molecular crowding, system biology, bioinformatics

The wonderful complexity of biological systems is responsible for the emergence of life from the chemical world, but it is also the reason why it is so difficult to address living systems in simulations. As recently demonstrated by the tremendous efforts directed to the study of SARS-CoV-2, even a relatively simple biological unit, such as a virus, needs to be addressed from multiple point of views—both as a whole, to study processes on the scales of microns and times of micro-milliseconds, as well as deconstructed into its single parts at the molecular level (Agúndez et al., 2020; Durrant et al., 2020). From the point of view of simulations, this implies following *in silico* the fate of (or tens or hundreds of) billions of atoms over macroscopic time scales. This appears impractical at a first sight especially for the computation cost, which, considering for instance Molecular Dynamics (MD) simulations, can be roughly estimated as ∞ $N_{D}^{\alpha }\times N_{t}={\left(S/dV\right)}^{\alpha }\left(T/dt\right)$ where *N*_*D*_ and *N*_*t*_ correspond to the number of degrees of freedom and the number of timesteps needed to represent a system of size *S* for a simulation time *T*; d*V*, and d*t* represent the discretization levels in space^1^ and time, and α is the exponent for the polynomial scaling of the computation cost with size^2^. Therefore, the history of molecular simulations is strongly interlaced with that of computing hardware development, both tracing back to the more than 50 years ago. The exponential increase of computing system performances up to now has led to the possibility of addressing whole viruses or (portion of) cells at the atomistic level in simulations of hundreds of ns (Tarasova and Nerukh, 2018), while simulations of single proteins can extend over the milliseconds scale (Shaw et al., 2009).

However, at the moment fully atomistic MD simulations cannot access simultaneously macroscopic sizes and time scales large enough for a sufficient statistical exploration. Therefore, they are often coupled to techniques for evaluating thermodynamic quantities (typically free energy profiles) as in the original research paper by Bagherpoor Helabad et al. combining Langevin Dynamics (LD) with entropy evaluation to identify the DNA binding domains of the androgen glucocorticoid receptor, or in that by Sun and Kekenes-Huskey, where the Potential of Mean Force (PMF) calculation along the open-close transition of the Ca^2+^ binding protein S100A1 involved in the cardiomyocyte function is operated with Weighted Histogram Analysis Method (WHAM) combined the Born surface area continuum solvation. With similar aims, a number of different techniques to expand the conformational and phase space is used, as reviewed by Bowman and Lindert focusing on the skeletal troponin. In these studies, stochastic dynamics (e.g., Brownian dynamics, BD) are combined with Umbrella Sampling-like techniques or steered molecular dynamics (SMD) and Markov chain modeling, with the result of effectively enhancing the conformational sampling. Similarly, the Gaussian MD method accelerates dynamics using an external potential to push the system out of the local minima, as in the simulations of Mitchell et al., on CRISPR-Cas9 in the presence of base pair mismatches. Also frequent is the combination of atomistic simulations and enhanced sampling techniques with bioinformatic methods, as in the template-based peptide sorting and docking algorithm (Peptidock) with the aim of designing peptides to interfere with Protein-Protein Interactions (PPI) for therapeutic scopes, as reported by Wang et al..

Besides the need of extending the simulation scales, there are other more subtle reasons that call for the search of new simulation strategies beyond conventional atomistic MD. One is that the first-generation atomistic Force Fields (FF), developed and tested during the last nearly six decades, start now to show their deficiencies, precisely due to the achievement of the macroscopic scales in simulations. As highlighted in the Perspective by Melcr and Piquemal, one shortcoming is the lack of polarizability due to the use of fixed partial charges, which determines a suboptimal representation of hydrogen bonds and as a consequence a poor description of secondary and tertiary structures relative stability, especially when the long time scales and temperature variations come into play. Thus, a tremendous parallel effort to reparameterize atomistic FFs to include polarizability has been ongoing, as in the AMOEBA FFs.

The failure in reproducing effects involving electronic rearrangements was one of the main driving factors inspiring the development of the multiscale approaches. The idea of multiscale is to combine atomistic FFs (molecular mechanics MM) with a higher resolution method explicitly representing electrons and therefore employing quantum mechanics (QM) in different space regions of the same system (hybrid QM/MM simulations, also called “parallel multiscaling”), in order to improve accuracy only in those regions where it is necessary. These regions are easily identifiable for instance in enzymes, where the active site is localized, making it possible the simulation of reactions such as the synthesis of Polycaprolactone—Polyethylene Glycol co-polymers, realized by Figueiredo et al. by means of an interface between the Gaussian code for QM and the Amber code for MM. The authors, additionally, couple the QM and MM methods even in a “serial way,” i.e., performing FF-MD simulations of the entire protein (no QM part) and QM simulations of the active site only, to compare and pass structural parameters between each other. In fact, in hybrid QM/MM simulations, the bottleneck of the calculation is the QM part, which also determines the reduction of the timestep of simulation, and consequently of the whole run length, implying an extension of the size of the system addressable with respect to QM only methods at same accuracy, but not of the time-scale. Therefore, a very important issue to solve is the efficiency of the implementation, which is addressed in the Opinion by Bolnykh et al.. Here the authors discuss the implementation realized in the MiMiC code, by means of a multiple program-multiple data paradigm, which combines the flexibility of the so-called *loose coupling* performed through an input/output interface between two different codes for QM and MM calculations with the computational efficiency of a *strong coupling* typically implemented in single *ad-hoc* codes for QM/MM. Additionally, to improve the extension of time scales of simulations MiMiC implements efficient multiple-time steps algorithms. We remark that, while the hybrid schemes solve in principle also the problem of polarization, the accurate treatment of electrostatics remains a crucial issue even in QM/MM approaches, addressed in MiMiC with the fully Hamiltonian electrostatic embedding. The hybrid QM/MM approaches can be coupled to methods for sampling enhancement as shown in the Perspective by Casalino and Magistrato focusing on the mechanism of Eukaryotes spliceosome, where combinations with thermodynamic integration, free energy calculations, principal component analysis of trajectories and electrostatic analysis are reviewed.

In biological systems the idea of multiscaling, or multiresolution approaches emerges naturally, because of the intrinsically hierarchical organization of biological matter, in which different levels of organization are easily recognizable. For biopolymers, the first super atomic level is that of the residue. Accordingly, the most popular super-atomistic (Coarse Grained CG) models are those based on a residue level representation. MARTINI and SDK FFs use, in fact, a slightly higher resolution (several 1-to-5 beads per residue) and explicit CG models for the solvent. This brings speed up the simulations of 200 to 400-fold with respect to atomistic ones, due in part to a direct reduction of *N*_*D*_, in part to the possibility of increasing *dt*, allowed by a the elimination of higher vibrational frequencies of the system, a secondary consequence of coarse graining. In practice the reduction of resolution operates a coarse graining both in the space and time domains, allowing the simulation of slow and extended processes like the budding of membrane and formation of lipid droplets, as described in the Opinion by Zoni et al.. MARTINI is among the more standardized CG FFs, and is often used in multi-scale approaches combined with atomistic simulations and e.g., homology modeling, as in the study by Glass et al. on the structure, function, and clustering of voltage gated sodium channels, or embedded within a flexible docking protocol to supplement atomistic rigid docking between proteins and nucleic acids, as this paper by Honorato et al. reporting a modified version of HADDOCK code.

A further considerable reduction of computational cost is obtained with CG implicit solvent models, especially those with simplified parameterization. Alfonso-Prieto et al. review the atomistic-CG “hybrid” (parallel) approaches based on a Go-like models, applied to G-Proteins Coupled Receptors, and show that these models can be used in combination with homology modeling and docking techniques, to dramatically improve the predictive power of binding affinity of ligands, especially due to the inclusion of flexibility of the whole complexes at low computational cost. Similarly, Delfino et al. use a Cα based minimalist model to address the large conformational changes of calmodulin upon Ca^2+^ binding/release, setting up a simulation paradigm that combines serially CG with atomistic representation and path searching, morphing, and minimum action path techniques, extendable to all switching proteins. D'annessa et al. review how atomistic and CG simplified representation such as the network models (EN) can be combined with docking algorithms, Monte-Carlo and MD possibly associated to enhanced sampling techniques (SD, WHAM, PMF) and implicit solvent treatments, focusing on applications to design peptide drugs to interfere with PPI.

A crucial point when considering CG approaches is related to the parameterization strategies. Besides the already mentioned simplified models (EN, Go-like, and minimalist) parameterized based on reference structures, parameterization strategies involve either bottom-up approaches based on higher resolution models or higher level theories (also called “physics based” or “*ab initio*”) usually involving the match of forces or energy surfaces, or top-down strategies (also called “knowledge based” or “data driven”), which incorporate experimental data, generally of different origin (thermodynamic, structural, vibrational). There is an ambivalent case: the “statistics based” parameterization, in which sets of structural data of any origin (measured or calculated) are used through Boltzmann Inversion (BI)-related procedures to fit the model parameters. The latter approach in particularly preferred when CG simulations are used to evaluate thermodynamic properties, because BI is the expression of thermodynamic consistency with the dataset. Oprzeska-Zingrebe and Smiatek show with a theoretical analysis that many subtle effect may arise at the bulk level in the evaluation of thermodynamic properties and equilibrium constants, depending on the specific choice of the size of the CG bead and its location, which therefore must be chosen very carefully. This is especially true when the coarse graining is pushed at very low resolution, e.g., a single bead per molecule or domain, sometimes called meso-scale (MS) level, often used to represent the crowders in the cell cytoplasm. Ostrowska et al. nicely review the recent literature of the crowded environment representations, which, incidentally, are usually “parallel” or hybrid multi-scale representations, since the system of interest, typically a protein, is represented at a higher resolution level than the crowders. The authors highlight the effects purely due to confinement, those due to the crowders shape or to the detail of the surface. A similar MS model decorated with CG beads is used by Brancolini and Tozzini to represent bio-functionalized metal nanoparticles designed as anti-aggregating therapeutic agents in degenerative diseases due to amyloidogenic proteins.

Clearly, the possible combination of different resolution and different sampling or parameterization methodologies are limited only by the researchers' creativity. For instance, Kandzia et al. use a MS level network model as external biasing potential for replica exchange atomistic MD (replicas differing by the level of bias) to study the slow motion and mechanism of action of the Hsp90 chaperone of yeast, giving an original example of parallel multi-scaling. Pezeshkian et al. give a perspective on their methodology that matches a continuum-like representation of the membrane with the particle-like representation. Their model represent the membrane by a dynamical triangulation including elasticity and the effect of membrane protein or inclusions, which can modify the elasticity and curvature, dynamically changing the parameters it via a Metropolis algorithm. The model parameters are calibrated using both atomistic and CG (MARTINI), with which the model is fully compatible, thanks to a back-mapping algorithm. The multi-scaling approach is also perfectly suited to represent the chromatin, the system in which the hierarchical structural organization is most evident. In particular, compaction-decompaction transitions are events triggered at the level of the nucleosome by chemical changes in the histone proteins, and reflect on the macroscopic level through a process where electrostatics plays a major role. Electrostatics also play a role in maintaining the delicate balance, which keeps the DNA relatively compact, yet accessible for the transcription and duplication. Bendandi et al. review the methods used to simulate these processes, involving all scales from atomistic to MS, and using several methodologies from MD to MC, implicit electrostatics, statistical, and mathematical modeling and analyses (e.g., topological and fractal models). The multi-scale approach is combined with the mathematical knot theory also by Rosa et al., using an inter-disciplinary approach to analyze the paradox of packing-entangling and accessibility of DNA.

In the course of the last decades the low-resolution models have evolved, and it has become clear that the combination of top down and bottom up-strategies in their parameterization can produce model with accuracy comparable or exceeding that of atomistic FFs, especially in the evaluation of thermodynamic properties. In the review by Orellana, the theme of cross-validation of *in vitro* and *in silico* is addressed, showing that the best way to tackle the complexity of live matter is a multi-disciplinary combination of enhanced sampling simulation techniques and path sampling methods applied to multi-scaling approaches mixing simplified models as EN with atomistic representation and experimental as CryoEM. The application focus is here on the switching proteins, ubiquitous, and difficult to address due to large conformational changes. However, a similar need for inclusion and cross-validation of models by means of experimental data emerges in the MS models for the cytoplasm, where, as shown by this brief report of by Kompella et al., standardized data about the composition in mass, size and diffusivity and inter-crossing relations between the cell elements are needed to set up a model for eukaryotic cells accurately reproducing the crowding effects.

Indeed, elements from system biology must be included when the level of simulation scales toward that of the cell. Widely used approaches in this case are those of Kinetic Master Equations (KME) connecting a set of cell elements. KME is used for instance in the representation of the whole complement cascade of the immune system illustrated in the Opinion by Zewde, where the vertices of the network are proteins, NAs and other cell components, and the kinetic parameters are evaluated through BD, within a “serial” coupling between particle-based and system biology methods. Similarly, Thornburg et al. address the processes of replication, transcription, and translation of a minimal synthetic cell, using atomistic data and genomic information for the parameterization. The model is able to predictively account for details such as the ribosomes production and activity. This should be considered a step forward in the representation of an entirely *in silico* cell.

The interdisciplinary character of multiscale approaches emerges clearly from the panoramic view on the methods illustrated in this collection, enriched by the contributions of the participants to the Workshop Multiscale Modeling from Macromolecules to Cell^3^ (CECAM Lausanne Feb 4-6 2019) organized by us and by which this collection was inspired. It is apparent that we are currently witnessing the historical moment in which the bottom-up computational approaches rising from the atomic and molecular level, and the top-down experimental methods, from the macroscopic level, meet at the mesoscale, where new possibilities of discovery and comprehension are enabled. Finally, before closing, we would like to comment on COVID19, the severe respiratory syndrome caused by the SARS-CoV-2 virus. COVID19 continues to unexpectedly test many of the cross-disciplinary and multiscale approaches discussed in this collection (Swiderek and Moliner, 2020) with many ongoing efforts from this community aiming to understand viral mechanisms of action (Zhao et al., 2020) as well as identify possible drugs and vaccines (Casalino et al., 2020). The urgency of the COVID19 situation has led to a unique combination of private-public worldwide coordination of governments, industries, and academies offering computing resources (Zimmerman et al., 2020) and sharing of methods, models, and data^4^. Although this terrible disease has not been defeated, yet, the incredibly rapid and coordinated worldwide research effort can already been considered a successful example to follow.

## Author Contributions

All authors listed have made a substantial, direct and intellectual contribution to the work, and approved it for publication.

## Conflict of Interest

The authors declare that the research was conducted in the absence of any commercial or financial relationships that could be construed as a potential conflict of interest.

## Footnotes

^1^*dV* is related to the resolution at which the system is treated. For instance, for the atomistic representation one can consider an average inter-particle distance of 1.5 Å leading to *dV* of the order of 10 Å^3^. For non MD techniques, *dt* can be substituted with a parameter describing the precision of phase or conformational space sampling.

^2^This is usually between 1 and 2, but may depend on the used model.

^3^Multiscale Modeling from Macromolecules to Cell, workshop website https://www.cecam.org/workshop-details/241.

^4^Public sites collecting data on SARS-CoV-2 https://pubs.acs.org/doi/10.1021/acs.jcim.0c00319, https://covid.molssi.org.
